# Women’s perceptions with use of Implanon^®^ contraceptive device at a primary healthcare facility in KwaZulu-Natal

**DOI:** 10.4102/hsag.v28i0.2016

**Published:** 2023-10-20

**Authors:** Lucky N. Mgobhozi, Gugu G. Mchunu, Pretty Mbeje

**Affiliations:** 1 Department of Nursing, Faculty of Health Sciences, Walter Sisulu University, uMthatha, South Africa; 2 Department of Nursing, Faculty of Science, Agriculture and Engineering, University of Zululand, eMpangeni, South Africa; 3 Faculty of Health Sciences, Durban University of Technology, Durban, South Africa; 4 Department of Nursing, Faculty of Health Sciences, University of KwaZulu-Natal, Durban, South Africa

**Keywords:** Implanon^®^, contraception, perceptions, family planning, primary healthcare

## Abstract

**Background:**

Early 2014, subdermal contraceptive implant was introduced in South Africa, Implanon^®^ NXT, aiming to expand the method mix, increase effectiveness and availability to long-acting contraceptives. The initial uptake was extremely high, but concerns have been raised with treatment failure and high number of removals reported.

**Aim:**

The study focuses on describing women’s perceptions with use of Implanon^®^ at a primary health care (PHC) facility in KwaZulu-Natal.

**Setting:**

This study was conducted at a selected primary health care (PHC) facility in KwaZulu-Natal.

**Methods:**

A quantitative, descriptive study design was used. Through purposive sampling, a sample of 60 women from 15 to 50 years old were recruited. Data were gathered through a structured questionnaire and analysed using SPSS 24 software.

**Results:**

Study findings show that slightly above half of respondents, 32 or 58.1% expressed satisfaction towards the implant, 20 or 40.9% had stopped using the implant as a result of its major implications. It was found that an edge above half of respondents continued using the implant 28 or 50.9%, while close to half had abandoned it (27 or 49.1%). Some respondents reported that they were experiencing heavy menstrual bleeding and low sex drive as serious unwanted side effects forcing them to stop using Implanon^®^.

**Conclusion:**

Side-effects and poor screening, counselling and support are major reasons for early removal. It is imperative to develop an effective screening tool and to re-train healthcare workers on Implanon^®^ NXT.

**Contributions:**

This article contributes to increase awareness of women’s perceptions about Implanon^®^ contraceptive.

## Introduction

In developing countries, including South Africa, implants have proved to be convenient, highly effectiveness and have a long duration of action (Jacobstein 2018:18–37). Implanon^®^ NXT, a long-acting subdermal contraceptive implant, first came into use in 2014 in South Africa as the government sought to expand contraceptive choice (Jonas et al. 2021:2; Krogstad et al. 2021:384). The South African Demographic and Health Survey (SADHS) (2017) reported high levels of uptake with use of modern contraceptive methods with 58% using a variety of contraceptives. Statistics on use of Implanon^®^ in South Africa show that in 2017, insertions rose to over 175 000 between 2014 and 2015 reporting period. However, this momentum was short-lived, and a decline of 50% was recorded in between 2014 and 2015 and further declined by 73% between 2014 and 2015 (Adeagbo et al. 2017:822; Beesham et al. 2021:41). The questions on the efficacy of Implanon^®^ are well documented. For instance, Beesham et al. (2019:750) investigated women’s perceptions for wanting to have the hormonal implant removed at an urban reproductive health clinic in KwaZulu-Natal. The findings of the study revealed that the South women sought Implanon^®^ removal as a result of side effects such as bleeding problem (15.8%), weight gain (5.8%), loss of libido (1.7%), headache (4.2%), dizziness (3.3%) and pain or numbness in the arm (1.7%), and more than a half (57.1%) reached 3 years duration and requested reinsertion. However, the study revealed the fact that some women who were experiencing side effects of Implanon^®^ discontinued using it. This study described women’s views regarding the use of Implanon^®^ as a contraceptive method at a selected primary healthcare facility at the uMgungundlovu district in KwaZulu-Natal Province. This study was conducted to understand the gaps and reasons for removal of Implanon^®^.

### Conceptual framework

The health belief model was used as the conceptual framework guiding this study. This theory is used to explain the main variables of the study and their interrelationships. Croyle (2005) defines the health belief model as a model that seeks to address problem behaviours that evoke health concerns. According to Potter and Perry (2005), the health belief model is described as a relationship between a person’s belief and behaviour. The health belief model constitutes three pillars, which include an individual’s perceptions, modifying factors and then likelihood of action.

The first and foremost pillar is ‘individual perception’ with Implanon, which entails the importance of contraceptive, perceived threat and perceived benefits of Implanon. Perceptions are mental frames of reference that can be based on either founded or unfounded statement or real or imagined events (Ayopo 2009). Perceptions also relate to people’s insight of an impression presented to the senses (Ayopo 2009). For this reason, individual perceptions could create the basis for reality. An individual perception is made up of three components, namely importance of contraception, perceived threat and perceived benefits.

The second pillar is ‘modifying factors’, which have significant impacts on the individual perceptions. These characteristics create influence on the personal perceptions. They are able to be adjusted or modified, which comprises three components, and these include demographic variables, interpersonal variables and situational variables.

The third pillar entails ‘likelihood to action’, which implies to the possibility to act upon the perceptions, barriers and influence made by modifying factors. However, certain individuals could take further actions based on potential negative consequences, knowledge given by health professionals and confidence to take their actions. This covers perceived barriers to actions, cues of action and self-efficacy. The health belief model was used to guide the study and assisted in the formulation of questionnaire. Therefore, study results will involve key concepts emanating from this model.

## Research methods and design

### Study design

This quantitative study used a descriptive research design to describe women’s perceptions regarding the use of Implanon^®^ as a contraceptive method in a selected primary healthcare facility at uMgungundlovu health district in KwaZulu-Natal. This design was utilised to gain a clear understanding and describe perceptions of women with use of Implanon contraceptive. Using the descriptive design, a highly structured, detailed statistical analysis of the profile, demographic data of participants was conducted (Brink, Van Der Walt & Van Rensburg 2018).

### Study setting

The study was held at a selected primary health care (PHC) facility under uMgungundlovu health district. The majority of the clients found at the community healthcare centre were middle-income class and at least had attended high school educational background. According to the Department of Health, KwaZulu-Natal (2019), the selected community health clinic serves as a 24-h comprehensive PHC service.

### Study population and sampling strategy

The study deployed a purposive sampling and identified (*n* = 60) women voluntary who met the inclusion criteria of this study (Brink et al. 2018). The population for this study was women who were using Implanon^®^ as a contraceptive method. The inclusion criteria for the study targeted women who were using Implanon^®^ as a contraceptive method at the selected primary healthcare facility. Additionally, female patients aged from 15 to 45 years who had Implanon^®^ for a period that exceeded 6 weeks or others that had discontinued the use of Implanon^®^, also among as well as female patients who had prematurely removed the implant before the Implanon^®^’s 3-year expiry, were included in the study. In addition to the stated criteria, the women who were present at the clinic at the time of the visit were considered for the study. The exclusion criteria included: female clients that have inserted Implanon^®^ for less than 6 weeks, mental healthcare users, pregnant women, clients in the emergency departments, labour wards and antenatal wards.

In order to establish the sample size, the target population was considered (Gray, Grove & Sutherland 2021). The researcher consulted a statistician to assist in determining the appropriate sample size in order to draw meaningful conclusions. As data collection was done over a period of a month, to calculate the sample size, the population estimated at ± 267 of women attending the clinic for Implanon issues and more was divided by the total number of months in a year, which is 12, in order to estimate the number of women who might be in the clinic on average in a month. This brought the number of each client seen at the clinic to 22.3. The researcher was working towards achieving a number of 60 as a sample size. The number of women who had the Implanon^®^ device inserted was 61, and thus, the sample size was determined to be 60. The sample was deemed appropriate as Gray et al. (2021) recommended that in quantitative studies, a smaller sample size can be 50.

### Data collection

Data for this study were collected through a structured questionnaire. A structured questionnaire is an instrument with questions that are decided by the researcher in advance (Brink et al. 2018). A structured questionnaire, with closed-ended questions, was developed by the researcher together with two supervisors in this study. The questionnaire was developed from health belief model. The structured questionnaire used concepts derived from the health belief model. Using the questionnaire was ideal for this study as it allowed the researcher to easily engage the respondents, thus achieving a much high response rate (Gray et al. 2021). Data were collected using ordinal rating scale that was quantified at the data analysis stage. The data collection tool was pre-tested with two respondents to assess reliability, and no alterations were deemed necessary to affect its usage. During the pre-testing phase, no data were gathered and no respondents were included in the study. Data were collected in June 2017 at a community health centre.

Identified respondents from uMgungundlovu District were engaged in response to the questionnaire at a selected primary healthcare clinic. Once a client arrived at the healthcare facility, the researcher first ascertained if the client had Implanon^®^. If the client confirmed, the researcher, supported by the clinic manager’s office requested the client to sit in the consultation room for data collection. In line with ethical standards, the client was informed about the aim of the study, and issues like confidentiality, harm, justice and voluntary participation were explained before the respondents were asked if they consented to participate in the study voluntarily. After signing consent forms, the data collection process commenced. The data collection process for each respondent took approximately 20–30 min, and thereafter, the respondents deposited their answer sheets in an enclosed container. The data collection procedure took a month to reach the required sample size.

### Instrumentation

This study adopted structured questionnaires. The researcher modified a readily available perceptions questionnaire divided into different sections in order to enable the processing of the data. The structured questionnaire was designed based on health belief model, which makes it more reliable, because it has been used before. The structured questionnaire was developed in English. All the questionnaires were coded for anonymity. The questionnaire contained only closed-ended responses that were included. The researcher consulted the statistician and computer expert, as well as supervisors, to sort through any errors or inaccuracies with the tool that was used. Questionnaires are a quick way of obtaining data from a large group of people and respondents enjoy a high degree of freedom in completing the questionnaires.

The structured questionnaire comprises sections A–D, with Section A that entails demographic information: This contains the respondent’s age, religion, marital status, occupation and level of education, employment status and previous method of contraception. The aim of utilising this data was to detect whether there was a connection concerning the demographic information of Implanon^®^ users with perceived barriers or enablers. Section B involves individual perceptions: This section contained of continuation with Implanon, perceived threat of falling pregnant, perceived susceptibility and perceived benefits. The respondents were asked if they still want to continue using Implanon and specify their perceptions to threat, susceptibility and benefits. Section C: Modifying factors: This section consisted of interpersonal variables and situational variables. Therefore, respondents had to specify whether their partners were aware that they were using Implanon^®^ and which healthcare professional were the most in contact with using Implanon^®^. Section D: Likelihood of contraception behaviour. This section comprises the items of perceived barriers and cues to the action. The respondent had to specify their perceived barriers and what health practitioners do to improve the knowledge gap.

### Validity and reliability

Validity was achieved in this study because the instrument was checked by supervisor and statistician for scrupulous verification in terms of its ability to measure the concepts being examined. To ensure reliability, a pilot study will be conducted on two respondents using the structured questionnaire designed for this proposed study prior to conducting the main data collection.

### Data analysis

In order to come up with descriptive statistics, SPSS 24 software was utilised to analyse data. The results obtained were presented using tables, charts and graphs. In order to ensure the credibility of the results, the data were analysed with the supervision and guidance of the research supervisor and statistician.

### Ethical consideration

Ethical approval to conduct the study was obtained from the University of KwaZulu-Natal Ethics Committee and the Department of Health. All necessary ethical measures were taken until informed consent was obtained from all the participants. All participants who were below the age of majority that is (less than 15 years) were excluded from not participating in the study as outlined by the guidelines for ethical practice in South Africa. Having excluded minors below 15 years, consent was only sought from minor participants above 15 years who, according to South African ethical guidelines, are permitted to autonomously give consent (Naidoo 2012). Measured to guarantee the confidentiality of respondents was taken by employing coded questionnaires instead of respondents’ real names. In addition, the respondents were informed that they had the autonomy to withdraw their participants at any time without attracting any penalty or prejudice as the study was of a voluntary nature. Right to freedom of choice and right to quality, justice, human dignity and protection against harm were adhered accordingly (Creswell & Creswell 2022).

Ethical clearance to conduct this study was obtained from the Biomedical Research Ethics Committee of the University of KwaZulu-Natal (No. BE604/16).

## Study findings

### Socio-demographic variables

The socio-demographic information of respondents included their age, marital status, religion, occupation and level of education. The results of the study are presented in Table 1.

**TABLE 1:** Demographic results of the population sample (*N* = 60).

Demographic characteristics	Variables	%
Age	15–20 years	9.2
	21–30 years	52.7
	31–39 years	34.5
	≥ 40 years	3.6
Marital status	Not married	72.8
	Married	21.8
	Divorced	3.6
	Widow	1.8
Religion	African tradition	34.6
	Muslim	1.8
	Christian	61.8
	Hindu	1.8
Occupation	Self-employed or Part-time	41.8
	Permanently employed	21.8
	Unemployed	23.6
	Still studying	12.8

*Source*: Mgobhozi, L.N., Mbeje, P.N. & Mchunu, G., 2017, ‘Exploring women’s experiences and perceptions on the use of Implanon as a contraceptive method in a selected primary health care facility in KwaZulu-Natal’, Masters thesis, University of KwaZulu-Natal, Durban.

## Individual perception

### Continuation with Implanon^®^

The study findings show that women’s perceptions varied when women were asked if they are still willing to continue using Implanon^®^. More than half of the respondents responded with a ‘Yes’ that they still use the implant for 31 (51.7%), whereas almost half responded with a ‘NO’ as to using the implant for 29 (48.3%).

### Perceived threat: Perceptions with side effects

The findings of the study show that out of 60 respondents who were using Implanon^®^, most of them, that is 34 (56.6%) indicated that they experienced two or more side effects, 8 (13.3%) experienced other side effects that were not provided on the list of side effects. Another 5 (8.3%) of the respondents indicated that they experienced menstrual bleeding. Related to the side effects of Implanon^®^, 5 (8.4%) of the respondents indicated that they had experienced weight gain, while others reported an unusual low sex drive, that is 4 (6.7%). Other side effects that included no response 2 (3.3%), insomnia 1 (1.7%), moods 1 (1.7%) were reported by the respondents. A number of side effects that were not on the list of side effects were mentioned by the respondents. These include dizziness, feeling cold, body pains, severe headache, loss of memory, weight loss, pregnancy, unusual and erratic menstrual periods, no menstrual period, vomiting and sweating.

### Perceptions with use Implanon^®^

Regarding women’s perceptions on using Implanon^®^, the results indicate that most of the respondents, that is 27 (45.5%) disagreed with the belief that the contraceptive is life-threatening when it is lost and moves inside the body, 17 (29.1%) agreed and 15 (25.4%) were not sure.

Additionally, most of the respondents, that is 33 (54.5%) disagreed with the belief that using Implanon^®^ can lead to a body’s vulnerability to critical illness, becoming weary or developing chronic ailments, 16 (27.3%) were not sure while 11 (18.2%) agreed.

### Perceived susceptibility with the Implanon^®^

The results revealed that 60 respondents believed that Implanon^®^ had side effects and risks for them. However, most of the respondents, that is 47 (78.2%) disagreed with the practice of having unprotected sex when one has Implanon^®^, 8 (12.7%) agreed and 5 (9.1%) were not sure. However, the research found that 31 (50.9%) of the respondents disagreed with the notion that Implanon^®^ use was associated with serious side effects, risks or illness, 20 (32.7%) were not sure and 10 (16.4%) agreed. The results further showed that some women think Implanon^®^ can disappear in their body, 26 (43.6%) of the respondents disagreed, while 23 (38.2%) were not sure and 11 (18.2%) agreed. Lastly, the results revealed that the majority of the respondents, that is 37 (61.8%) disagreed with the belief that having Implanon^®^, a foreign body is terrifying and uncomfortable, 20 (32.7%) agreed and 3 (5.4%) were not sure.

### Perceived benefits

#### Reasons for using Implanon^®^ implant

The reasons why women use Implanon^®^ were consistent with their perceptions on the effects of the contraceptive. The findings show that the majority of the women, that is 35 (58.2%) decided to use Implanon^®^ because they were attracted to its permanence, 12 (20.0%) used the implant after getting recommendations, 10 (16.4%) chose to use the contraceptive because there is no follow-up after getting the implant, 2 (3.6%) believed the contraceptive was associated with fewer side effects and 1 (1.8%) responded that it was a reason other than those mentioned.

#### Satisfaction with Implanon^®^

The results of the study indicate that less than the half, that is 23 (41.8%), were not satisfied with using Implanon^®^, whereas 16 (29.1%) are satisfied with the implant and 16 (29.1%), are extremely satisfied with the use of the implant.

### Modifying factors

#### Informed their partner’s that they use Implanon^®^

Analysis evidenced that the majority, that is 41 (69.1%) affirmed telling their partners about using Implanon^®^ and 19 (30.9%) responded that their partners did not know they were using the Implanon^®^.

#### Acquire with the new Implanon^®^

Analysis shows that the half of the respondents, that is 28 (50.9%) were informed at the clinic by healthcare professionals, posters and pamphlets; two thirds of the respondents 16 (29.1%) found out from peers who are friends, partners and neighbours, whereas 7 (12.7%) were informed through media (radio, television, newspaper, social network and internet). Lastly, 3 (5.6%) found out about the implant at work, either from other colleagues or occupational health nurses. None found out at school from educators and from school health teams.

## Likelihood of contraceptive behaviour

### Decision to use Implanon^®^ for 3 years

The drivers of using Implanon^®^ for the entire 3-year duration varied from person to person. However, the majority of the respondents, that is 34 (56.4%) indicated that they would use the implant for the 3-year duration, 21 (34.5%) disagreed that they would have the implant for the entire 3-year duration and 5 (9.1%) indicated that they might use the implant for the 3-year duration.

### Awareness with the new Implanon^®^

Regarding women’s awareness with the implant, the results show that half of the respondents, that is 31 (50.9%) gained knowledge about the implant through their nearest health facility, healthcare workers, health awareness posters and pamphlets, 17 (29.1%) of the respondents indicated that they got to know about the implant from word of mouth, through friends, partners and neighbours. Few of the respondents, 8 (12.7%) learnt about the implant through the media (radio, television, newspaper, social network and internet). Lastly, 3 (5.6%) of the respondents indicated that they learnt about the implant at work, from colleagues or occupational health nurses. Remarkably, none found out at academic institutions, that is, educators and from school health teams.

### Understanding with use Implanon^®^

The study findings showed as little as one third of respondents, that is 54 (90.0%) believed that anyone can have Implanon^®^ inserted, 4 (7.3%) believed that only young women with no children could receive the implant and 1 (1.8%) believed that only women within the child-bearing age could use the implant (Table 2).

**TABLE 2:** Responses to those respondents with the Implanon^®^ (*N =* 60).

Findings	Variables	%
Side effects	Weight gain	9.6
	Menstrual bleeding	41.8
	Mood changes	10.9
	Acne	10.4
	Insomnia	6.9
	Loss of sex drive	16.8
	Pains/redness on the site of insertion	3.6
Continuation to use Implanon^®^	Yes	50.9
	No	49.1
Informed their partner	Yes	69.1
	No	30.9
Women’s satisfaction with the implant	Not satisfied	41.8
	Satisfied	29.1
	Extremely satisfied	29.1

*Source*: Mgobhozi, L.N., Mbeje, P.N. & Mchunu, G., 2017, ‘Exploring women’s experiences and perceptions on the use of Implanon as a contraceptive method in a selected primary health care facility in KwaZulu-Natal’, Masters thesis, University of KwaZulu-Natal, Durban.

### Partner’s perceptions with Implanon^®^

The results show the majority, that is 41 (69.1%) of the respondents had made their partners aware of their use of Implanon^®^, while 19 (30.9%) of the respondents indicated that they had not informed their partners that they were using the Implanon^®^, as mentioned in Table 2.

### Perceptions, knowledge and barriers of Implanon^®^

The results of the study indicate that one third of the respondents, that is 52 (87.3%) were affirmative that they understood Implanon^®^ owing to the assistance of healthcare providers, while 8 (12.7%) did not get the same privilege. It was also found that 49 (81.8%) were well positioned to inquire and get answers about this contraceptive implant from the healthcare providers, while 10 (16.4%) were not able to ask and get relevant answers and 1 (1.8%) were uncertain.

Among 60 respondents, just over half, that is 37 (61.8%) possessed knowledge about the side effects of the Implanon^®^ for the first 3 months of receiving it, while 20 (32.7%) of the respondents indicated that they did not have any knowledge of the Implanon^®^ side effects and 3 (5.5%) were not sure. More than a half, that is 35 (58.2%) affirmed that they experienced barriers or issues that might cause them to stop using Implanon^®^, while less than two thirds, that is 22 (36.4%) responded that they had none and 3 (5.5%) expressed that they were not sure. It was also evidenced that more than two thirds, that is 33 (54.5%) affirmed that they had thought to discontinue using Implanon^®^ in favour of another contraceptive, while just less than half 25 (41.8%) said they did not think of changing, and a minimum of 2 (3.6%) were not sure.

## Discussion

### Side effects of Implanon^®^ contraceptive and management

The findings demonstrate that a majority of women experienced side effects associated with using Implanon^®^ contraceptive device. The side effects varied, but the dominantly reported ones were vaginal bleeding or periods disturbances, loss of body weight, headaches and pains, memory loss or disturbances, insomnia and loss of sex drive. The study participants reported that each time they reached the clinic with menstrual bleeding or periods, only contraceptive pills were at their disposal that were helping them temporarily with the symptom, but after completion of treatment the bleeding issue was returning. The perceptions on bleeding patterns on women were changed and improved after the use of mifepristone and doxycycline prescription (Siyoum et al. 2017:6–16). However, the study findings also revealed that there was no positive change in the subsequent bleeding patterns. Contrary to these findings, a qualitative study on reasons for early removal of Implanon among users in Arba Minch town, Gomo Goffa zone, South Ethiopia: a phenomenological approach (Mesfin et al. 2020:1–7) found that the most frequently occurring side effect of Implanon was heavy irregular bleeding; however, there was no treatment mentioned to correct this side effect. Thus, vaginal bleeding was the main reason for the removal of Implanon in many users. As Jacobstein (2018:16), menstrual bleeding issue is a common problem to all women that use the implant and is a significant challenge that can be modified through treatment. However, the treatment has been found to work over a short period, thus only solving the problem temporarily (Beesham et al. 2019:751). The study establishes that despite an increase in reports about side effects of the implant, there is very little education offered to clients regarding the subject. As a result, women who experience side effects easily discontinue the implant before its duration lapses. As Nega, Abera and Tadele (2021:12) highlight, counselling during insertion of contraceptive implant is important and should be adequately given to women before they start the insertion. Department of Health (2017) also emphasises the importance of contraceptive counselling and the provision of initial and continuing contraceptive care. Effective counselling and screening can improve contraceptive continuation even though there may be side effects that clients experience (Tadesse et al. 2017:2).

### Discontinuation of implant

The study findings also show that 49.1% of women stopped using Implanon^®^, whereas 50.9% continued. The main causes for discontinuation cited were side effects (34.5%), where menstrual bleeding (41.8%) was found to be the predominant factor for discontinuation, followed by loss of sex drive, with 30.9% and 25.4% acne. Among the women, menstrual bleeding stood out and even among those, they were required to seek medical assistance from the clinic regularly. The World Health Organization (WHO) (2019) also showed that the continuation rate was very low in developed countries; there were 55.4% women that continued to use Implanon^®^ in the first 2 years after insertion and 47.5% were also those using Norplant**^®^** within a 2-year duration in developing countries. Vaginal bleeding was the most significant side effect leading to premature discontinuation of these methods (Rabopape et al. 2019; Siyoum et al. 2017). These findings were similar to those found in a study conducted by Beesham et al. (2019), where it was reported that menstrual disturbance is the most common reason for discontinuation, with headache, acne, weight gain and desired for pregnancy as the other reasons for removal.

### Perceptions of women using Implanon^®^

The study focal point was women using Implanon^®^ from 15 to 49 years of age, including those that have discontinued. Harries et al. (2019) also indicate that age-related issues can be a factor that impact the contraceptive practices. The study’s finding shows that a majority of women who use Implanon^®^ were between the ages of 21 and 30 with the percentage of 52.7%. Similarly, the results found by Adeagbo et al. (2017) on the prevalence use of contraceptive by young people between the ages of 15 and 24 years, where the results indicated that the usage of contraceptives in young people was 52.2%. This can be linked with the findings shown by UNFPA (2018), stating that South African girls and women between 15 and 49 years of age use contraceptives at a higher rate of 60%, compared to sub-Saharan countries with an average of 20%. In contrast, this result shows that most women that took initiatives to attempt this new contraceptive were between 21 years old and 30 years old. However, the young adolescents using the implant were low in number (9.1%). According to Pillay, Morroni and Pleaner (2017), it was found that there were few adolescents using new contraceptives implant among all age groups. Moreover, a qualitative study conducted in Limpopo Province about contraceptives shows that 60% of girls were not utilising healthcare services, where it was found that healthcare workers denied them access to contraceptives.

## Recommendations

These recommendations are presented as proposed methods and interventions that will assist to improve standards in the service provision of Implanon^®^ contraceptive devices. Therefore, these interventions were made based on the study findings in this research investigation:

- Prioritise early screening and counselling to reduce cases of adverse events with the use of Implanon^®^ among women. Most adverse events reported in this study were cases that can be prevented earlier only if proper screening and counselling were done initially such as proper history taking, and performing investigations required in screening and educating. Additionally, women should be counseled about side effects to be expected initially and should be informed about possible treatments to correct side effects.
- Schedule booking for removal must be conveniently rendered as a service and not be very far in cases where clients are having adverse effects thereof and they decide to change. At least clients should be free to remove the implant any day they want without any pressure to continue to use the implant.
- All healthcare providers especially nurses working in PHC facility, should receive inservice training on the use of Implanon^®^. The client should receive all the relevant help and information at the clinic during one consultation at a healthcare facility. Therefore, this will also allow staff to work as a team when dealing with various conditions and in solving side effects or issues brought forward by clients.
- Effective screening tools should be in place and developed specifically for Implanon^®^ that will allow healthcare practitioners to easily adopt and understand the entire requirement needed for this implant.
- Imperative research investigations should be conducted broadly to explore Implanon^®^ contraceptive method and even involve more health facilities and clients. Investigation should involve healthcare providers as to identify issues and experiences they might have regarding this new contraceptive method.

## Study limitations

The study sample was at a very small scale and could have been extended to a larger scale to explore the findings. Because of a small sample, the study findings cannot be generalised to provincial and national findings.

## Conclusion

Overall, the perspectives of the women suggest that better measures should be taken to deal with side effects, as in most cases, women stop using Implanon^®^ because of side effects; otherwise, they use Implanon^®^ because it lasts longer, and most are extremely satisfied with the use of Implanon^®^. Most respondents are extremely satisfied with the assistance they get from healthcare facilities. It was found that most women heard about Implanon^®^ from their peers and from clinics. Among the issues that they had were side effects, commonly menstrual bleeding, weight gain, acne and loss of sex drive. Many removal reasons were mainly because of the side effects and poor screening, counselling and support. This method requires an effective screening tool and to retrain healthcare workers about Implanon^®^. Furthermore, policies and guidelines should be implemented correctly regarding the management of side effects and the care rendered by healthcare practitioners.
